# MicroRNAs in cancer: biogenesis, biomarkers and therapeutic strategies

**DOI:** 10.3389/fmed.2026.1801949

**Published:** 2026-05-07

**Authors:** Nivedita Rawat, Neelesh Babu, Aabid Hussain, Abhaya Shikhar Panwar

**Affiliations:** 1 Department of Biotechnology and Microbiology, School of Allied Sciences, Dev Bhoomi Uttarakhand University, Dehradun, Uttarakhand, India; 2 Department of Genomic Sciences and Systems Biology, Cleveland Clinic Research, Cleveland Clinic, Cleveland, OH, United States; 3 Department of Botany, S.G.S. Degree College, Dehradun, Uttarakhand, India

**Keywords:** cancer, inhibitors, microRNAs, miRNA mimics, oncomirs, therapeutics, tumor-suppressor miRNAs

## Abstract

MicroRNAs (miRNAs) are identified as critical regulators in cancer biology, imparting a significant impact on tumor development and metastasis. miRNAs are identified as small, highly conserved, endogenous, non-coding RNA chains of 20–28 nucleotides. They target messenger RNAs and use to regulate their gene expression post-transcriptionally and modulate an extensive number of cellular mechanisms, for instance cell cycle control, regulation of stress response, and programmed cell death. It is evident from the studies that dysregulation of miRNA through genetic mutation, processing defects, and altered expression may cause various human diseases, including cancers, which underscores the role of miRNA either as oncogenic or as tumor-suppressing genes. miRNAs have been found as an emerging crucial regulatory tool for prognostic, diagnostic, and therapeutic applications for numerous malignancies. This review delves into highlighting significant aspects of miRNAs from the perspective of cancer, with respect to their biogenesis, dysregulation, and their function in cancer metastasis. Moreover, several miRNAs as oncogenes and tumor suppressor genes have been discussed along with different miRNA-based therapies for suppression and treatment of cancers.

## 1 Introduction

MicroRNAs (miRNAs) are among the shortest endogenous, highly conserved non-coding RNA molecules, consisting of about 20 to 28 nucleotides (1). miRNAs are concerned to the gene expression regulation through complementary binding to the sequences on messenger RNAs (mRNAs), following mRNA degradation or translation inhibition (2). Evidently, cell differentiation, proliferation, metabolism, and cell death are the key cellular processes regulated by miRNAs, through post-translational regulation, hence contributing to the maintenance of cellular homeostasis (2).

The foremost miRNA that was found in the nematode *Caenorhabditis elegans* (*C. elegans*) in 1993 by the Ambros and Ruvkun research groups was Lin-4. It was formerly identified as the genes accountable for the development of the temporal region of *C. elegans* larvae by regulating the lin-14 gene (3). Later on, studies indicated that Lin4 was identified as a non-coding RNA that does not code for protein (4). The discovery of lin4 as a non-coding RNA was followed by the detection of a new miRNA, let-7, found in *C. elegans*, where it was known for the negative regulation of the mRNA by interacting with its 3′ untranslated region (3’UTR) (5). Afterwards, a number of miRNAs have been identified possessing an important role in biological processes across a number of species, including humans, animals, and plants etc., (6). However, it is also evident that dysregulation of miRNAs can disrupt the biological processes leading to certain disease development, including cancer.

Globally, Cancer poses a major health challenge, becoming a leading cause of morbidity and mortality, accounting for approximately 19 million new cases and 10 million deaths each year (7). The first significant confirmation connecting miRNAs to cancer was identified by Croce and colleagues in 2002. They recognized the miR-15 and miR-16-1 cluster as key miRNAs positioned at chromosomal part 13q14, a region which is frequently found deleted in chronic lymphocytic leukemia (CLL). CLL is a cancerous condition characterized by the accretion of mature B lymphocytes (B cells) in the bone marrow, lymph nodes and blood (8). Studies have proved that miRNA can work as either oncogenes or tumor suppressors in a variety of cancers. miRNA sequencing and profiling have provided convincing evidence related to miRNA dysregulation and cancer initiation and progression, suggesting that specific miRNAs hold significant potential as biomarkers for tumor identification, diagnosis, and prognosis (9).

In the present review, we discuss the role of miRNA in causing cancer, either acting as an oncogene or tumor suppressor gene, and its regulation. Moreover, the different cancer-based therapies for the treatment or control of the disease are discussed.

## 2 miRNA biogenesis and regulatory control

**Figure 1:**
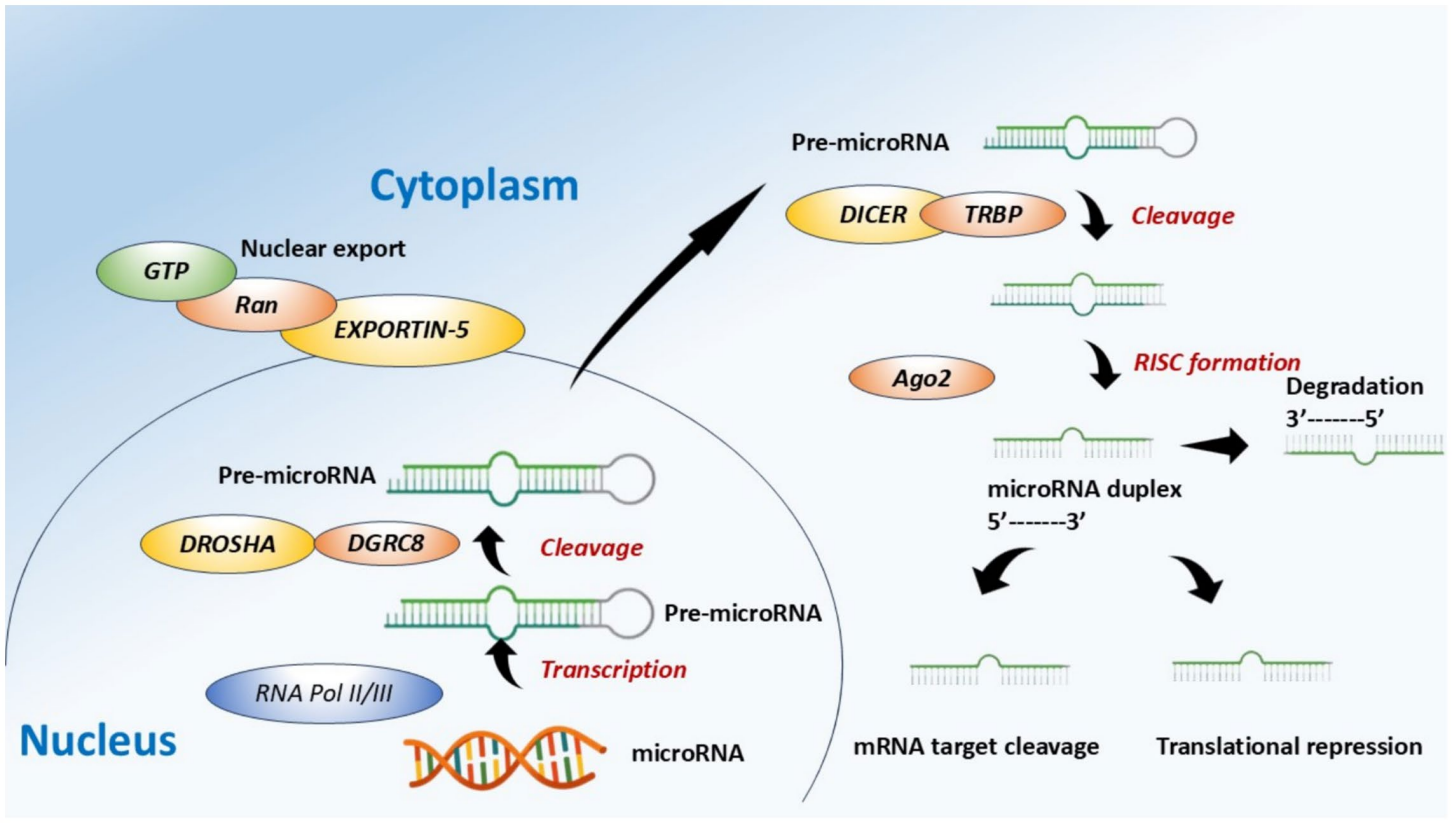
Schematic representation of the biogenesis of miRNA and its regulation.

miRNA biogenesis is a two-step pathway, comprising both nuclear and cytoplasmic processes (Figure 1). The synthesis of miRNA starts in the nucleus, where miRNA genes are transcribed by RNA polymerase (RNAP) II/III into primary miRNAs (pri-miRNAs) (8). Pri-miRNA is a long transcript that consists of a characteristic stem-loop structure. This pri-miRNA is then changed by its cleavage in the nucleus, leading to the formation of precursor miRNA (pre-miRNA) with the help of a microprocessor complex, consisting of a double-stranded RNA-specific ribonuclease known as Drosha and its cofactor DGCR8 (10, 11). The pre-miRNA, about 70 nucleotides in length, is then exported for its further processing from the nucleus toward the cytoplasm with the assistance of Exportin-5/Ran GTP complex (10). Afterwards, in the cytoplasm, the RNase-III enzyme known as Dicer processes pre-miRNA by removing the hairpin loop, yielding a short, 21–28 nucleotide double-stranded RNA duplex (11). This duplex structure has two types of strands: the first one is a guide strand (which is a mature miRNA strand), and the second one is a passenger strand (which is found complementary to the guide strand) (8, 11). The passenger strand gets degraded, but the mature miRNA strand is loaded onto the RNA-induced silencing complex (RISC), which guides the complex to complementarily target the mRNAs. The miRNA-mediated silencing occurs through mRNA decay or is determined by the degree of miRNA match to its target sequence (12).

However, it is apparent that miRNA biogenesis is a tightly regulated and synchronized multi-level process involving transcriptional regulation, nucleocytoplasmic processing by Drosha and Dicer, nuclear export, and RISC complex loading. Mutations in microprocessors like Drosha and Dicer can interrupt the pathway for miRNA biogenesis, forming aberrant expression profiles of miRNA. Mutations in *DROSHA* and its cofactor *DGCR8* have also been recognized as oncogenic carriers in Wilms tumor, which is known as the prevalent kidney cancer in children (13). In addition, *DGCR8* dysregulation has been observed in several cancers, and mutations in the *DGCR8* gene have been found linked to the growth and development of thyroid cancer (14). Furthermore, Drosha expression is often found downregulated in a variety of cancer types, resulting in reduced miRNA levels and an ineffective gene silencing mechanism (15, 16). Therefore, mutations of these regulatory genes can lead to insufficient miRNA processing, ultimately impacting target gene expression and increasing cancer risk.

Additionally, defects in export proteins such as XPO5 can damage nucleocytoplasmic export of pre-miRNA, resulting in reduced levels of miRNAs in the cytoplasm, leading to interruption in the miRNA-mediated gene regulation (17). Dysregulation at any of these steps may result in abnormal miRNA expression, contributing to several diseases including neurological disorders, metabolic conditions, and cancer (18). In recent decades, the widespread dysregulation of miRNA in cancers highlighted the significant role of miRNAs in tumorigenesis, metastasis, and cancer development (19). The primitive evidence of miRNA’s contribution in human cancer was observed in the year 2002, where it was noted that deregulation of miR-15a and miR-16-1, cluster gene at chromosome 13q14 was found to be connected with the B-cell chronic leukemia (B-CLL) (20). Here, the target genes miR-15a and miR-16-1 are involved in the progression of the cell cycle and the regulation of apoptosis, thus acting as tumor suppressors. Deletions or structural abnormalities in the 13q14 genomic region resulted in a reduction in miR-15a/16–1 expression, in that way driving the initiation and progression of chronic lymphocytic leukemia CLL (21).

## 3 miRNAs in tumorigenesis

miRNAs act as oncomirs (promoters of cancers) and onco-suppressors (suppressors of tumors). Alterations in genomic miRNA such as apmplifications, deletion or translocation can result in abnormal miRNA expression. miR-15 and miR-16 cluster in chronic lymphocytic leukemia (CLL) marked one of the earliest pieces of evidence connecting micro RNA to cancer, recognizing miRNAs to have an important function in the regulation of the gene (21). miR-15 and miR-16 act as tumor suppressors by targeting genes involved in cell cycle progression and apoptosis. Evidently, the loss of genomic region in any way can lead to reduced expression of miR-15 and 16, hence contributing to the rise of CLL (21). Additionally, single nucleotide polymorphisms (SNPs) have been recognized in the genes for miRNA-146a, miRNA-149, miRNA-196a2 and miRNA-499 (8).

Dysregulation of miRNAs is closely linked with changes in the signaling pathways which are found to govern cancer development and progression. For instance, miR-9 is downregulated in oral squamous cell carcinoma, where its restoration induces cell cycle arrest and suppresses proliferation through inhibition of CDK6 and Cyclin D1. Interestingly, miR-9 exhibits context-dependent roles, as in glioma it can target multiple genes such as COL18A1 and PTCH1, thereby promoting proliferation and cell cycle progression (16, 21).

Depending on the target genes, miRNA can function as Tumor-causing miRNAs which are overexpressed in cancer cells, hence contributing to tumor development by attacking the genes responsible for the regulation of cell cycle, programmed cell death (PCD), and DNA repair. Some important groups of oncogenes include miR-21, miR-181a, miR-221/222, and miR-155, involved in metastasis and proliferation of neoplastic cells (22). On the other hand, a few miRNAs function as onco-suppressors by downregulating oncogenes or genes that promote cancer development. The downregulation of tumor-suppressive miRNAs is frequently observed in cancer, resulting in the upregulation of oncogenes and accelerated tumor development (Table 1).

**Table 1:** miRNAs unusually expressed in cancers.

Cancer type	Predominantly upregulated miRNAs	Predominantly downregulated miRNAs	References
Chronic lymphocytic leukemia	miR-21, miR-155	miR-15, miR-16, miR-29b, miR-34, miR-143, miR-145, miR-223	(88–90)
Breast cancer	miR-10, miR-21, miR-22, miR-27, miR-155, miR-210, miR-221, miR-520	Let-7, miR-7, miR-9, miR-17, miR-31, miR-200 family, miR-205, miR-335	(91, 92)
Lung cancer	miR-17–92 cluster, miR-21, miR-106, miR-155	Let-7 family, miR-1, miR-7, miR-15, miR-29 family	(93–95)
Prostate cancer	miR-221, miR-222	miR-15/16 cluster, miR-101, miR-127, miR-449	(96, 97)
Hepatocellular carcinoma	miR-17–92 cluster, miR-21, miR-143, miR-224	miR-1, miR-101, miR-122	(98, 99)
Colorectal cancer	miR-17–92 cluster, miR-21	miR-34, miR-127, miR-143, miR-145, miR-342	(100, 101)
Lymphoma	miR-17–92 cluster, miR-155	miR-143, miR-145	(102–104)
Ovarian cancer	miR-214	miR-34, miR-200 family	(105, 106)
Melanoma	miR-221, miR-222	Let-7a, miR-34	(107, 108)
Gastric cancer	miR-21, miR-27a	miR-143, miR-145	(109, 110)

Some tumor suppressor miRNAs include let-7 family, miR-34 family, and miR-15a/16–1 cluster (23).

### 3.1 Oncogenic miRNAs

#### 3.1.1 miR17-92 cluster

miR17-92 is the foremost miRNA cluster discovered to have oncogenic properties, which is expressed in several cancers, especially in human hepatocellular carcinoma (HCC) (24). In humans, the miR17-92 cluster is located on chromosomal region 13q31.3. The cluster consists of 6 miRNAs, such as miR-17, miR-18a, miR-19a, miR-20a, miR-19b-1, and miR-92a-1 (21). Mammals encode a related paralog cluster, recognized as miR-106a-363 and miR-106-25 on chromosome number 10, containing a similar set of miRNAs (25). The miR17-92 is observed to be upregulated in various cancers, including lung cancer, lymphomas, and several other tumors, consistent with its conventional role (16). There are two mechanisms responsible for the upregulation of miR17-92 in cancer. The foremost one is chromosomal amplification of the 13q31 locus, found to be present in different types of lymphomas (26). Another one is transcriptional activation of the cluster by oncogenic transcription factor (cMyc). Here, cMycbinds directly to the genomic region upstream of the miR-17-92 cluster, acting as a powerful transcriptional activator of its expression (27). However, this function is performed by MYCN, a protein that is highly associated with c-Myc in the case of neuroblastoma (28). Additionally, it is also found that E2F transcription factors are capable of enhancing miR-17-92 expression (29). In addition, the miR-17-92 cluster forms part of an intricate regulatory network that helps balance cell growth and survival. Experimental evidence from animal models has demonstrated that functionally, the miR-17-92 cluster makes a contribution in tumor development and progression by promoting cell proliferation, cell invasion, and inhibition of apoptosis (programmed cell death). Studies have shown that in transgenic mice, overexpression of miR-17-92 in combination with c-Myc overexpression accelerated the development of B-cell lymphoma (30). On the contrary, mice lacking this cluster show reduced tumor formation and increased apoptosis, establishing its role in tumorigenesis. Additionally, the miR-19 family has been recognized as an important pro-survival factor through its capability to suppress PTEN, a tumor suppressor gene (31).

#### 3.1.2 miR 21

**Figure 2:**
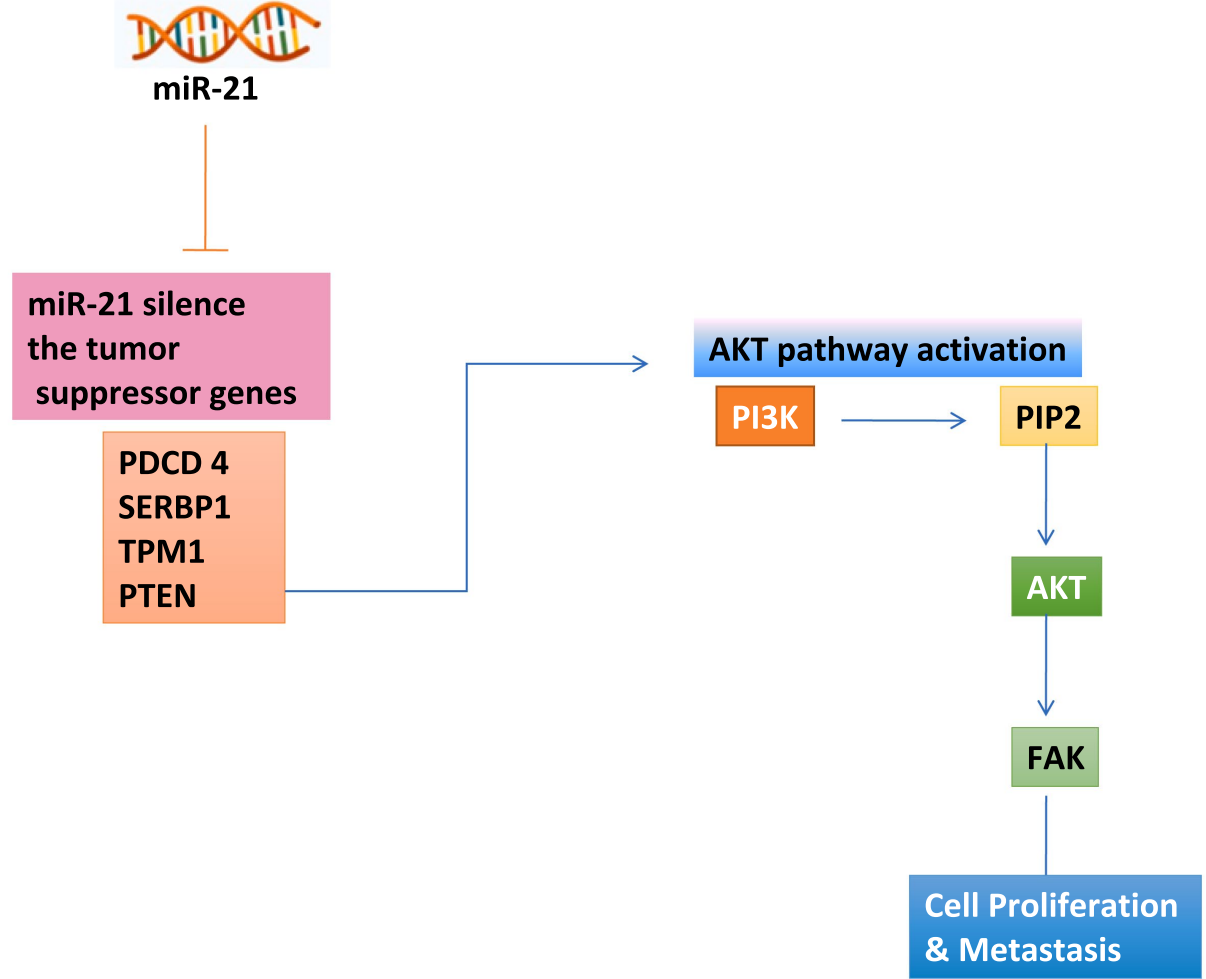
Mir-21 mechanism of regulation in cell proliferation and metastasis.

The role of miR-21 in oncogenesis was first highlighted in cases that were analyzed for abnormal expression levels of miRNAs in the disease glioblastoma, which is an aggressive and malignant brain tumor (32). miR-21 is highly upregulated in normal animal cells; subsequently, its overexpression has been observed in numerous cancers, including ovarian, colon, pancreatic, and prostate cancers (33). miR-21 upregulation supports tumor cell survival by acting as an antiapoptotic factor and suppressing the genes involved in promoting apoptosis (32). Evidently, miR-21 contributes to cancer development by downregulating various tumor suppressor genes by binding to 3’UTR transcripts of mRNA. miR-21 functions as an oncogene by silencing key tumor suppressors: (1) programmed cell death 4 (PDCD4), concerned in regulating cell death and blocking cell proliferation, (2) Maps 1 (SERBP1), a gene involved in translation regulation and mRNA stability, (3) TPM1 (Tropomyosin-1), which stabilizes actin filaments and regulates cell morphology. The downregulation of TPM1 by miR-21 increases cell motility and invasiveness, and (4) PTEN (Phosphatase and tensin homolog) controls cell endurance and expansion by inhibiting PI2K/AKT signaling pathway. Suppression of these tumor suppressor genes by miR-21 promotes cell transformation, growth of tumors, invasion and metastasis. PTEN and phosphoinositide 3-kinase (P13K) works in coordination, regulating intracellular levels of PIP3 which is a ket modulator of the Akt signaling pathway. PTEN dephosphorylates PIP3, hence maintaining the cellular balance, while PI3K converts PIP2 into PIP3 (Figure 2) (16, 34). When miR-21 suppresses PTEN at the translational level, PIP3 accumulates, leading to hyperactivation of the Akt pathway. This regulatory process hence stimulates downstream signaling cascades that enhance cell growth and survival. In addition, loss of PTEN function is linked to enhanced activity of FAK (Focal Adhesion Kinase), promoting cell proliferation and metastasis by upregulating matrix metalloproteases (Figure 2). The loss of PTEN expression due to miR-21 activity results in activation of this pathway, which promotes cell proliferation, survival, and resistance to apoptosis (Figure 2) (34, 35).

Furthermore, TPM1 which is an actin binding protein plays a significant role in maintaining the integrity of cytoskeletal and regulating anchorage-independent growth. Hence, its suppression by miR-21 is believed to induce changes in cytoskeletal, supporting neoplastic transformation and metastasis (35).

Experimental studies in animal models have confirmed miR-21’s role in various cancers. In a study done in mice, overexpression of miR-21 resulted in aggressive lymphomas, whereas miR-21 knockout mice led to a reduced tumor burden (36). Prominently, these tumors became reliant on continuous miR-21 expression, as turning off the miR-21 causes rapid tumor regression, likely due to increased apoptosis. These findings advocate that inhibition of miR-21 could be a promising therapeutic strategy in cancers driven by its overexpression.

#### 3.1.3 miR-155 and B-cell integration cluster (BIC)

BIC was formerly recognized as a common retroviral incorporation site in chicken lymphoma cells caused by avian leukosis virus (AVL). The BIC gene was later identified as the precursor to miR-155. Subsequent studies demonstrated that in a chicken model, this locus promotes MYC-mediated lymphomagenesis (37). This discovery marked miR-155/BIC as a promoter of tumor development, and its overexpression was constantly observed in numerous tumors, such as breast, lung, colon, and most importantly in B-cell lymphomas (38). The research with miR-155/BIC driven by a B-cell specific enhancer was found to have elevated levels of the mentioned miRNA causing polyclonal B-cell malignancies with little or no other genetic alterations in transgenic mice (39). Additionally, miR-155 is also connected to key cancer pathways as its expression is upregulated in mutant p-53 driven tumor invasion in breast cancer, hence contributing to tumor cell invasiveness (40). Additionally, miR-155/BIC also found its role in regulating immune responses, especially in B-cell maturation and T-cell differentiation. Therefore, miR-155/BIC can be considered as a potent biomarker for cancer recognition and prognosis, as well as its potential therapeutic target because of its widespread overexpression in tumors and its connection to its aggressive tumor behavior (41, 42).

#### 3.1.4 miR191/425 cluster

The miR-191/425 cluster is observed in numerous metazoans, which is situated in the 1st intron of the DALRD3 (DALR anticodon binding domain containing 3) gene on chromosome 3 (3p21.31) (43). This cluster encodes four mature miRNAs: miR-191-3p, miR-425-3p, miR-191-5p, and miR-425-5p. While both miR-191 and miR-425 promote breast cancer cell proliferation, miR-191 alone supports erythroid enucleation. miR-191 was first studied in mice and later detected in humans, initially in the HL-60 leukemia cell line (44). It is considered an oncogenic miRNA, overexpressed in at least 16 cancer types, including breast, prostate, lung, pancreatic, liver, stomach, bladder, ovarian, esophageal, and hematological malignancies such as B-ALL and AML (43).

#### 3.1.5 miR221/222 cluster

The miR-221 and miR-222 cluster, situated on the X chromosome (Xp11.3 region), consists of two strongly related miRNAs: miR-221 and miR-222. These miRNAs are found to be upregulated in many human cancers, with high expression observed in hepatocellular carcinoma (HCC) (45). About 80% of HCC cases show noticeably elevated levels of miR-221 and miR-222, and it’s observed that, as compared to normal liver tissue (46). Both miR-221 and miR-222’s overexpression contribute to tumor progression, enhanced migration, invasion, and inhibition of apoptosis, imparting their oncogenic effect. This cluster is also found in circulating body fluids, where it can be considered as a biomarker for early recognition of cancer and disease monitoring, with promising avenues for clinical applications (47).

### 3.2 Tumor-suppressive roles of miRNAs

#### 3.2.1 miR-15/16 family

There are four members in this microRNA family, which function as tumor repressors. They are organized into two autonomous clusters: miR-15a/miR-16-1 and miR-15b/miR-16-2, where miR-15a/miR-16-1 is found to be situated at chromosome 13q14.3, and miR-15b/miR-16-2, at 3q26 (33). These four members consist of common 9-nucleotide regions that target the 3’UTR of the BCL2 mRNA, which blocks the cell death process by blocking the release of mitochondrial cytochrome-c that activates the caspase pathway. miR-15/16 family leads to the post transcriptional repression of BCL2, a key anti apoptotic protein (48). In chronic B-cell lymphocytic (CLL), the miR-15a/16-1 cluster is recurrently deleted, where its loss causes the dysregulation of BCL2, resulting in elevated BCL2 protein levels and increased resistance to apoptosis (48, 49). Therefore, the miR-15/16 family demonstrates tumor suppressive effects by regulating BCL2 by contributing to the activation of cell death pathways. However, beyond CLL, the miR-15/16 family has shown tumor suppressive functions in prostate, lung, colorectal, and breast cancers (48).

#### 3.2.2 miR-29 and miR-34

The miR-29 and miR-34 contribute a significant function in regulating cancer development, growth, and progression, acting as tumor suppressors. They both are found to be downregulated in different malignancies such as glioblastoma, neuroblastoma, osteoblastoma, lung cancer, colon cancer and blood cancer (50). The miR-29 and miR-34 families control the p53 tumor suppressor protein signaling axis for effective tumor suppression. miR-29 acts mainly as an upstream regulator enhancing p53 stability and activity while miR-34 act as downstream effector of p53. Their actions stabilize a coordinated system that amplifies p53 responses to cellular stresses (50).

**Figure 3:**
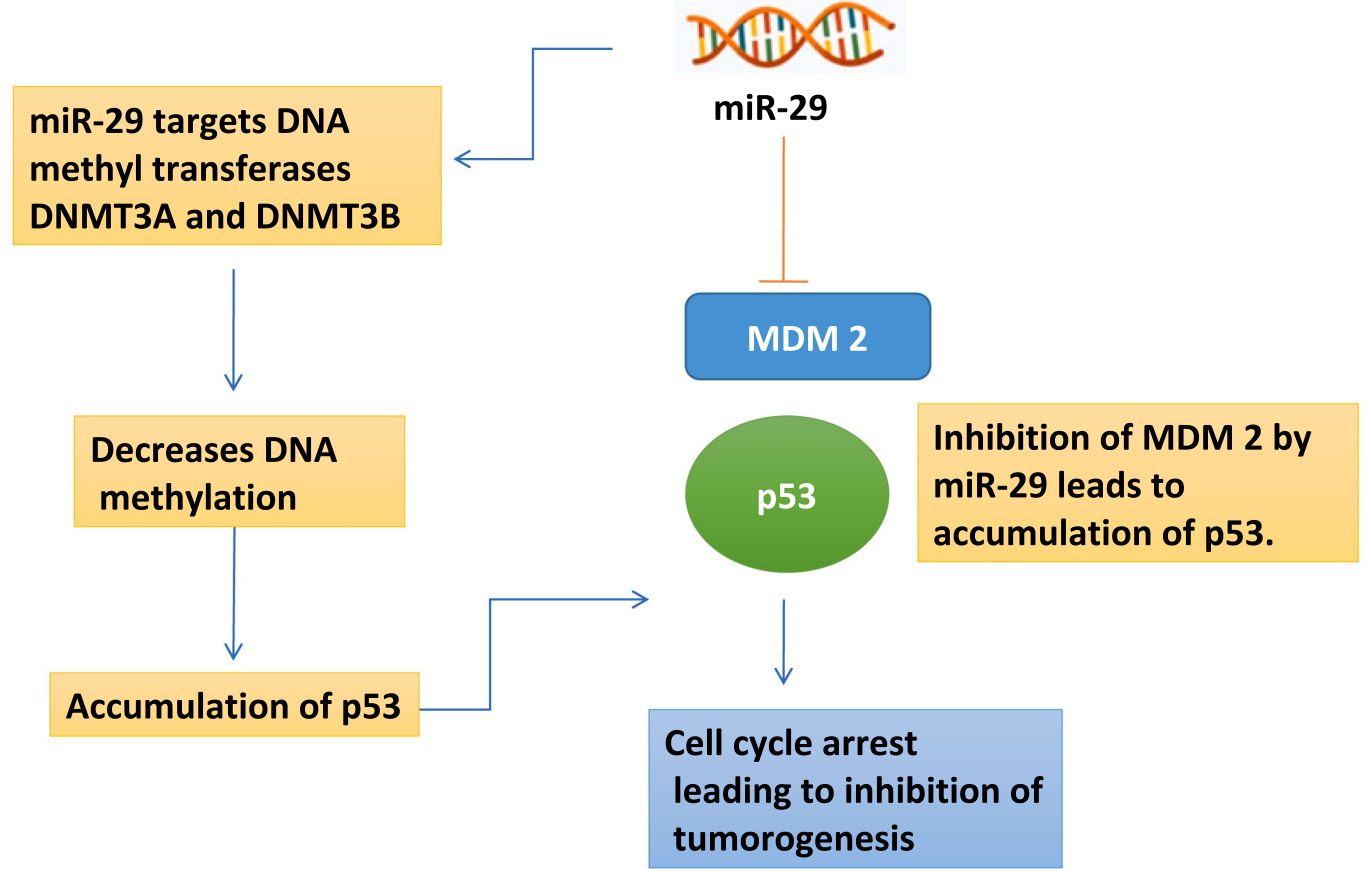
MiR-29 mechanism of regulation as tumor supressor.

Evidently, miR-29 regulates the p53 pathway indirectly by targeting upstream inhibitors and epigenetic modifiers. miR-29 suppresses proteins that negatively regulate the stability and function of p53, predominantly influencing pathways involving MDM2, responsible for ubiquitination and degradation of p53 (Figure 3). By reducing MDM2 activity, miR-29 promotes stabilization and accumulation of p53. Furthermore, miR-29 targets DNA methyltransferases such as DNMT3A and DNMT3B, leading to decreased DNA methylation and reactivation of tumor suppressor genes, including components of the p53 pathway (Figure 3) (50, 51). However, these DNMTs can also methylate and silence mi-R 29 gene promoters. Thus, when miR-29 levels are low, DNMT activity increases, leading to repression of miR-29 expression participating in an epigenetic feedback loop which can either maintain gene activation which is high miR-29 or stable silencing which is low miR-29, significantly influencing p53 signaling. (51, 52). Through these actions, miR-29 enhances the cellular environment required for optimal p53 function. MiR-29 regulates ovarian cancer, where it inhibits autophagy and reduces resistance to cisplatin via modulation of the FOX/ATG14 pathway (51).

**Figure 4:**
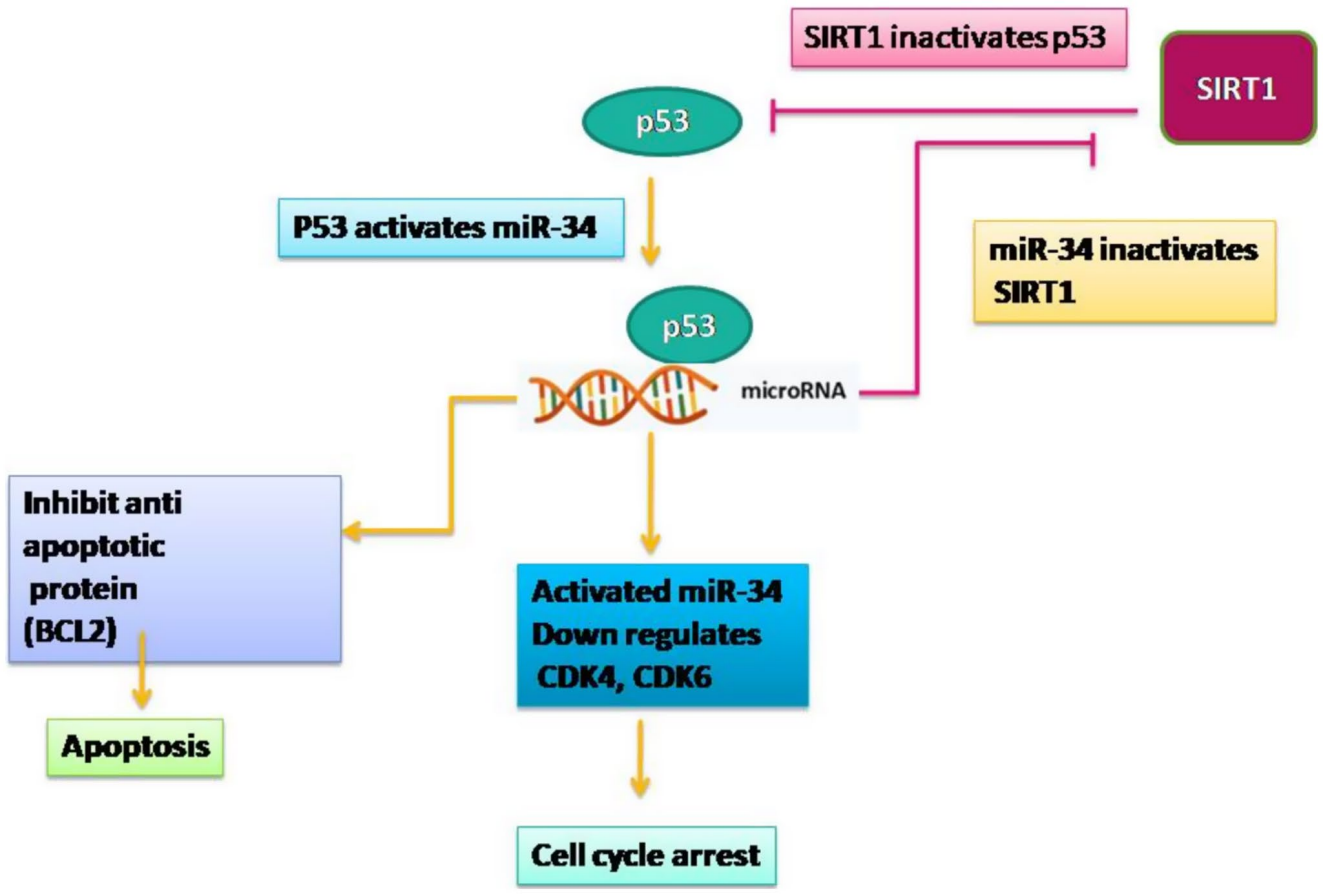
MiR-34 mechanism of regulation as tumor supressor.

Likewise, miR-34 functions as a tumor suppressor, influencing the drug-resistant mechanism of cancer cells. The miR-34 family includes miR-34a, miR-34b, and miR-34c, that are highly conserved and often function redundantly (52). miR-34 is transcriptionally activated by p53, where p53 binds to the promoter regions of the miR-34 gene, inducing their expression. miR-34 then mediates key tumor-suppressive functions of p53 by targeting multiple genes involved in cell cycle progression and survival. It downregulates critical regulators such as CDK4, CDK6, and Cyclin D1, leading to G1/S cell cycle arrest (Figure 4). Additionally, miR-34 promotes apoptosis by inhibiting anti-apoptotic proteins like BCL2. Another crucial function of miR-34 is the suppression of SIRT1, which normally deacetylates and inactivates p53. By inhibiting SIRT1, miR-34 enhances p53 acetylation and activity, thereby forming a positive feedback loop that strengthens and sustains p53 signaling (Figure 4) (16, 53). It is evident that low levels of miR-34 are connected to low chemotherapy response; however, re-establishment of miR-34 expression can enhance the sensitivity of resistant tumor cells, showing its potential as a therapeutic agent (52).

#### 3.2.3 Let-7 family

The let-7 miRNA family was at first recognized in *C. elegans*, regulating developmental timing (53). Let-7 plays a significant role in coordinating developmental stages and promoting cellular differentiation, and its dysregulation contributes to abnormal cell proliferation and behavior in cancer (16). It is a critical tumor suppressor, regulating oncogenic pathways, basically targeting the oncogene Ras and high mobility group AT-hook 2 (HMGA2). Ras is a GTP-binding signal transducer that provides signals from cell surface receptors to intracellular pathways hence controlling cell proliferation and tumor induction. Let-7 miRNA binds to Ras mRNA at the 3’UTR and represses the production of the Ras protein thus restraining Ras mediated cellular processes (16). In cancer cells, downregulation of let-7 leads to enhanced Ras activity and increased oncogenic signaling. Certainly, in several human cancers such as lung cancer, breast cancer, colorectal cancer, and others, reduced levels of let-7 have been persistently observed, confirming its role as a tumor suppressor (54).

Furthermore, the let-7 family also targets other oncogenes such as MYC, KRAS, and HMGA2, additionally confirming its function in restraining tumor growth. HMGA2 is a non-histone chromatin binding protein that modulates gene transcription by altering chromatin structure and it is highly expressed during embryonic development and in certain tumors. The let-7 family regulates HMGA2 by binding to its 3’UTR, leading to destabilization of its mRNA. Loss of let-7 mediated regulation results in the increase of HMGA2 expression hence promoting enhanced cell growth and proliferation (16, 54). This wide role of let-7 in regulating certain oncogenes and several key modulators like c-MYc, CDC25A, CDK6 and cyclin D2 makes restoring let-7 expression in tumors an attractive therapeutic strategy (27).

## 4 Role of miRNA in cancer

Angiogenesis, metastasis, and invasion are the major processes involved in tumor development. Angiogenesis is the creation of new blood vessels for the delivery of oxygen and nutrients and oxygen to the developing cancer cells. Vascularisation is achieved by inducing vascular endothelial growth factor (VEGF) and fibroblast growth factor (FGF). These factors stimulate endothelial cells to degrade the extracellular matrix leading to the proliferation, migration, and formation of new vascular structures (55). miRNAs have been known as crucial regulators of cancer development. Such as miR-126, which is primarily expressed in endothelial cells, and its dysregulation enhances angiogenic signaling by targeting a negative controller of VEGF and FGF known as Spred-1 (56). Likewise, miR-17-92 cluster was observed to be activated to promote tumor vascularisation in Myc-driven tumor 21, whereas miR-378 promotes tumor angiogenesis in ovarian cancer by downregulating anti-angiogenic molecules for instance activated Leukocyte cell adhesion molecule (ALCAM), and EH domain containing protein-1 (EHD1) (57). In addition, miR424, miR130a, and miR296 were also identified as modulators of angiogenesis by influencing the expression of key angiogenic receptors and inhibitors (58, 59).

Subsequent to angiogenesis, tumor development undergoes the process of invasion, a crucial step for metastasis, where the tumor cells go through the epithelial to mesenchymal transition (EMT), followed by malignancy. The EMT process facilitates accelerated migration and invasive potential in cancer cells; however, one of another transition, i.e., mesenchymal-to-epithelial transition (MET) is responsible for the metastasis outgrowth (59). EMT, which increases cell motility and invasion, is considered by the loss of epithelial markers like E-cadherin, which is found to be responsible for adhesion in normal cells. The significant signaling pathways, such as TGF-*β*, and critical transcription factors like SNAIL, ZEB, and TWIST help in regulating the epithelial to mesenchymal transition (60). Several miRNAs are observed to play roles in upregulation or downregulation of EMT and metastasis. The miR-192 family and miR-200 family prevent metastasis and epithelial breakdown by targeting signaling pathways such as TGF-β, thus suppressing EMT (61). Decrease in the amount of these miRNAs has been linked with an increased number of cases of breast and ovarian cancers. Conversely, miR-155 is found to be upregulated in various malignancies, where its expression is found to be induced by signaling pathways such as TGF-β/SMAD4 (62). Additionally, in neuroblastoma and breast cancer, miR-9 acts as a promoter of metastasis and is transcriptionally regulated by oncoproteinsn-Myc and c-Myc. These oncogenic transcription factors n-Myc and c-Mycbind to the miR9-3 locus to stimulate its transcription; the resulting overexpression of miR-9 directly leads to the downregulation of E-cadherin in breast cancer 8 (21). Whereas in colorectal cancer (CRC), miR-212 acts as a tumor repressor, which is found downregulated, due to hypermethylation of the promoter and genetic loss, and has been observed to inhibit CRC cell invasion and migration (63, 64). Meanwhile, some miRNAs such as miR-17, miR-21, miR-29a, miR-31, and miR-122 are overexpressed in CRC, contributing to their invasive nature *in vitro* and *in vivo* (59).

Despite the direct roles of miRNAs in influencing the signaling pathways, miRNAs also promote cancer progression through epigenetic regulation. Epigenetic mechanisms regulate miRNA expression primarily at the level of transcription by modifying the chromatin structure. The methylation of DNA occurs at CpG islands located in miRNA gene promoters, where DNA methyltransferases (DNMTs) add methyl group to cytosine residues, following to condensation of DNA and inhibition of transcription of miRNAs. Along with DNA methyl transferases (DNMTs), histone deacetylases (HDACs) also contribute to the epigenetic regulations in cancers (65). The example is miR-101, which inhibits a histone methyltransferase named Enhancer of Zeste Homolog 2 (EZH2), which is involved in gene silencing and linked to aggressive tumor phenotypes. miR-101 targets EZH2 and reduces cancer cell survival and metastatic capability, which demonstrates the function of microRNAs in shaping the wider epigenetics of cancerous cells (65, 66). The most noticed epigenetic targets of miRNAs are DNMT1 (maintenance methyltransferase) and DNMT3A/DNMT3B (*de novo* methyltransferases) residing to DNMT family. For instance, the miR-29 family targets openly DNMT3A and DNMT3B dropping DNA methylation and re-expression of tumor suppressor genes in lung cancer and hematologic malignancies (64). Additionally, miR-148a and miR-152 have been revealed to target DNMT1, resulting in decreased promoter hypermethylation and restitution of silenced genes in hepatocellular and gastrointestinal cancers (65).

Similarly, histone deacetylases (HDACs), regulate chromatin condensation and transcriptional suppression through histone deacetylation. miR-34a, targets HDAC1 and HDAC2, thereby influence cell cycle progression and apoptosis (65). miR-449, also suppresses HDAC1 expression and contributing to epigenetic reprogramming (65, 67). Furthermore, miR-1 and miR-206 have been reported to target HDAC4, affecting differentiation and tumor progression pathways (65), while miR-22 has been shown to regulate HDAC6, modulating cellular motility and stress responses (65).

## 5 miRNA-based therapies

The therapies based on miRNA can be classified into two major paradigms: (1) miRNA inhibition therapy, where the overexpression of the miRNA can be suppressed by antagonists. (2) miRNA mimics or replacement therapy: the normal function of a downregulated or dysfunctional miRNA can be rescued by the use of synthetic oligonucleotides. Here, the function of normal miRNA is mimicked by synthetic oligonucleotides known as miRNA agonists (67).

### 5.1 Inhibition therapy

The inhibition of tumor-causing miRNAs is achieved by miRNA sponges or anti-microRNA oligomers (AMO), miRNA masking antisense oligonucleotides, and antagomirs (67).

#### 5.1.1 Antagomirs and anti-miRNA oligomers

The synthesis of anti-miRNA oligomers or antagomirs having complementary sequences with endogenous miRNA is the most prevailing method to inhibit miRNA function. Due to their cognate function, these antagomirs are also known as antisense oligonucleotides (ASOs). They bind to the endogenous miRNAs, thus promoting miRNA degradation by preventing their processing by RISC complexes (68, 69). These antagomirs targeting oncogenic miRNAs can be considered for the treatment of diseases like cancer. Antagomirs or ASOs are single-stranded antisense oligonucleotides, 17–22 nucleotides in length, intended to bind precise mRNA followed by of its translational inhibition (70). Mechanistically, antagomirs bind to the mature miRNA guide strand through complementary base pairing. in view of the fact that, most mature miRNAs are integrated into RISC, whose functional core consists of Argounate proteins, inhibition characteristically occurs at the level of miRNA-loaded RISC complex. Antagomir binding intervene the miRNA seed region, thereby preventing recognition of complementary sequences within the target mRNA 3’UTR. This blockage distorts recruitment of RISC to target transcripts and consequently selectively deactivate RISC complexes containing the target miRNA (71). They are found to have blocking action on the upregulated oncogenic miRNA, which has a competent and enduring effect (71). In a study on pancreatic tumor cells, where ASOs were used targeting miR-21 and miR-221, leading to an elevated expression of the genes such as RECK, PTEN, and CDKN1B, that suppress of tumor, along with reduced proliferation and enhanced apoptosis (72). ASOs are also found to sensitize pancreatic ductal adenocarcinoma (PDAC) cells to gemcitabine, which is a chemotherapy drug, resulting in synergistic anticancer effects (73).

#### 5.1.2 Chemical modifications of antagomirs

**Figure 5:**
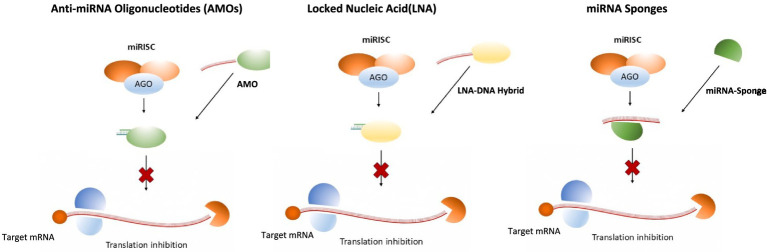
Schematic representation of major miRNA silencing approaches, including antisense oligonucleotides (AMOs), locked nucleic acid (LNA)-modified inhibitors, and miRNA sponges. The figure shows three approaches to inhibit microRNA (miRNA) function. Anti-miRNA oligonucleotides (AMOs) bind directly to mature miRNAs, preventing their interaction with target mRNA. Locked nucleic acids (LNAs) form highly stable duplexes with miRNAs, blocking their incorporation into the RNA-induced silencing complex (miRISC). miRNA sponges act as competitive decoys with multiple binding sites, sequestering miRNAs. All three strategies inhibit miRNA-mediated translational repression and restore target gene expression.

Various chemical modifications are employed to antagomirs to attain their complete delivery and stability, helping in moderating responses. Additionally, these modifications strengthen the stability of miRNA modulators against nuclease-mediated degradation in circulation. The modifications in AMOs include the addition of 2’-O-methyl (2’-OMe) and 2’-O-methoxyethyl (2’-MOE) groups at the 5′-end of the molecule and locked nucleic acid (LNA) (Figure 5) (74). A comparative analysis of the chemical modifications of Antagomirs is shown in Table 2.

**Table 2:** Comparative analysis of 2’-OMe, 2’-MOE and LNA modifications.

Parameters	2’-OMe	2’-MOE	LNA	References
Structure	Methyl group at 2′-OH	Methoxyethyl group at 2′-OH	2′-O,4′-C methylene bridge forming bicyclic structure	(21, 67)
Conformation	RNA-like conformation	Stronger RNA-like conformation and hybridization stability	Locked C3′-endo conformation with increased structural rigidity	(21, 67)
Resistance to nuclease	resistance to nuclease	Improved stability compared to 2′-OMe	Significantly enhanced stability due to conformational constraint	(21, 67, 76)
Binding affinity	Improved binding efficiency to mRNA	Superior binding affinity and target specificity vs. 2′-OMe	Markedly increased binding affinity to complementary RNA	(21, 67, 76)
Stability	Enhanced duplex stability	Greater hybridization efficiency	Strongly enhanced thermodynamic stability	(21, 67, 76)
Target specificity	High target affinity	Superior specificity to mRNA targets	Very high specificity due to strong base pairing	(21, 67, 76)
Toxicity	Low toxicity; suitable for therapeutic use	Efficient and therapeutically valuable with favorable profile	Requires careful optimization; high affinity may increase off-target risk	(21, 67, 76)
Clinical use	Widely used AMOs for miRNA inhibition; cost-effective	Valuable in therapeutic development; splicing redirection and translation inhibition	Demonstrated therapeutic efficacy in cancer models	(21, 67, 75, 76)

#### 5.1.3 2’-O-methyl group (OMe) modifications

Since the hydroxyl group at the 2′ end of nucleotides gets easily attacked by nucleases, consequently, modification of the 2′ end becomes the most important chemical alteration of the molecule. Here, the addition of OMe at the 2’end of the ASOs provide to their resistance to the nuclease enhancement with improved binding efficiency to mRNA. Synthetic ASOs incorporating 2’-OMe modification have been shown to inhibit miRNAs that have been upregulated in certain cancers. Specifically, miR-21 is a well-known oncomir that has been successfully targeted using 2’-OMe AMOs in human cell lines of breast cancer and glioblastoma (75). Nowadays, 2’-OMe-modified AMOs has become the most widely used tools for inhibiting upregulated miRNA function. They have also become well-suited for therapeutic applications due to their high target affinity, low toxicity, and cost-effectiveness (67).

#### 5.1.4 2’-O-methoxyethyl modification

As compared to 2’-O-methyl (2’-OMe) analogs, 2’-O-methoxyethyl (2’-MOe) modified antagomirs confirm the superior binding affinity and target specificity to mRNA. These chemically modified oligonucleotides have proven valuable tools in therapeutic development due to their efficiency in redirecting mRNA splicing and inhibiting protein translation (67). This modified oligonucleotide hybridizes with target RNA using the Watson-Crick base pairing and blocks the production of oncogenic miRNA by preventing translation of the mRNA transcript.

#### 5.1.5 LNA

LNA is a chemically improved class of antisense molecules. These molecules are characterized by a modification of the ribose sugar, where a 2’-Oxygen and 4’-Carbon are linked by a methylene bridge, creating a bicyclic structure. This linkage locks the sugar in a C3’-endo sugar RNA-like confirmation, providing increased stability and higher binding affinity (21). This structural confirmation constraint significantly enhances both the binding empathy and thermodynamic stability of oligonucleotides containing LNA, to the complementary RNA targets (76). In a study, it was identified that the inhibition of miR-133a using LNA showed curative importance in the case of treating osteosarcoma, basically in reducing cell invasion and improving patient outcomes. Furthermore, modified antagomirs, in addition to LNA or other chemical modifications, show prolonged half-life as compared to original miRNA mimetics. Research has shown that inhibition of miR-221 and miR-21 by means of modified antisense oligonucleotides (ASOs)resulted in elevated levels of expression of the target genes, including (CDKN1B) cyclin-dependent kinase inhibitor 1B, PTEN (phosphatase and tensin homolog), and reversion-inducing cysteine-rich protein with RECK. Additionally, miRNAs such as miR-16, miR-21, and miR-181a act as probable targets for cancer therapy of the lung by utilizing the AMOs. AMO-based inhibition of these miRNAs has been found to hinder tumor cell growth, increase cell death, and arrest cell cycle progression, offering a targeted strategy for improving therapeutic outcomes in lung carcinoma (67).

#### 5.1.6 miRNA sponges in cancer therapy

The miRNA sponge tool was brought to the knowledge in 2007 as plasmid constructs. miRNA sponges are engineered to contain several sites that are observed to bind to the seed region of target miRNA by complementary binding, enabling effective sequestration of that miRNA (77). The seed region is an important nucleotide sequence of the miRNA, which is known for the determination of its target specificity. After transfecting cells, this plasmid constructs transcripts that express high levels of RNA sponges that bind to and sequester a family of miRNAs sharing a common seed sequence, effectively blocking their activity (78). miRNA sponges consist of short nucleotide fragments exhibiting similar inhibition efficiency, owing to their competitive behavior. For long-period miRNA loss-of-function studies, sponge inhibitors have been engaged in there constitution of the bone marrow and cancer xenografts. miRNA sponges in cancer therapy act or mimic the downregulation of specific miRNAs that are dysregulated (67). In this one study, scientists measured the levels of microRNA-21 (miR-21) expression in the patient serum samples who reviewing who are suffering from non-small cell lung cancer (NSCLC) before evaluating the biological effects of miR-21 on A549 NSCLC cells. Through the use of miRNA sponge technology and A549 cell transfection, the research found that miR-21 might function as a stand-alone molecular biomarker for non-small cell lung cancer. Additionally, the results imply that modifying miR-21 or PDCD4, its downstream target, may be a unique therapeutic approach for non-small cell lung cancer treatment (67). Furthermore, miR-181 sponge was found to inhibit the whole miR-181 family in a cardiac cell line (79).

#### 5.1.7 miRNA masking

miRNA masking technology exercises single-stranded 2’-O-methyl (OMe) antisense oligonucleotides (modified) that bind within the 3’UTR sequence of the target mRNAs. The chemical modification of antisense oligonucleotides enhances their half-life in biological systems by increasing their stability and resistance to nucleases (80). By masking miRNA binding sites in mRNAs, the miRNA mask prevents miRNA binding to respective mRNA, enabling gene-specific regulation without affecting the miRNA regulation of other genes. miRNA masking has evolved as a hopeful approach in cancer therapy by specifically acting upon miRNA gene interactions, which are involved in controlling the critical processes in tumorigenesis.

## 6 miRNA mimics or replacement therapy

**Figure 6:**
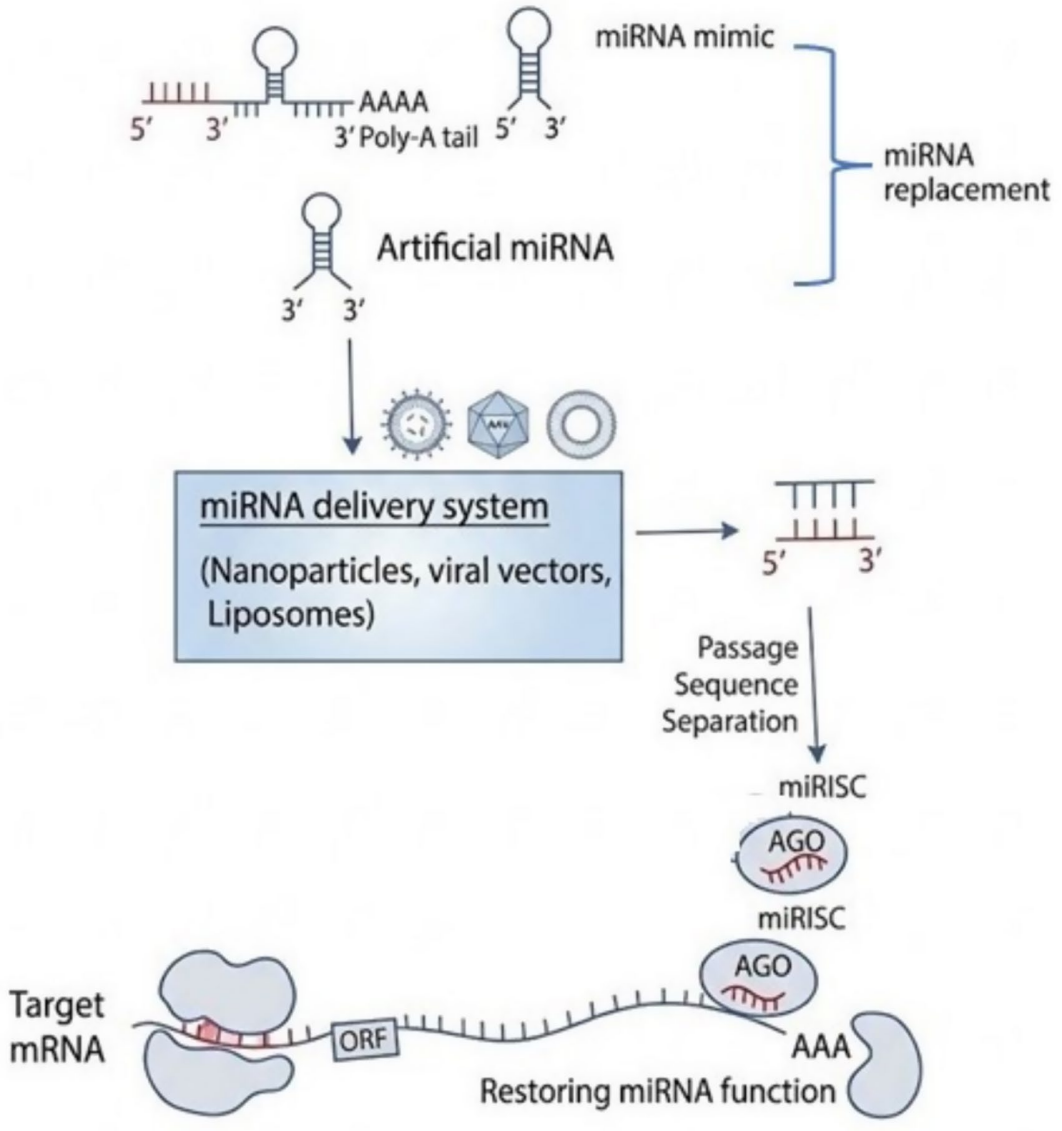
Schematic illustration of restoring miRNA function using miRNA mimic.

In replacement therapy, the purpose of a downregulated miRNA is recovered by using miRNA mimics that are chemically synthesized oligonucleotides. These miRNA mimics, when integrated, can re-establish the normal physiological action of the organism by mimicking the function of the down-regulated or under expressed miRNA (Figure 6) (21). The functioning of miRNA replacement technology involved the design of the active and guide strand of the miRNA mimic to imitate the miRNA nucleotide sequence that is maturely expressed in the cells. Upon incorporation, the active strand binds into the RNA-induced silencing complex (RISC), and the passenger strand is chemically modified to inhibit unintended interactions, ensuring the miRNA mimic acts specifically and functionally (81). Experimental studies demonstrated that miRNA let7, which was at first recognized as a switch gene essential for physiological development in *Caenorhabditis elegans*, was found to be downregulated in laryngeal cancer (21). Let-7 in wild-type cells suppresses the expression of the Rat Sarcoma (RAS) oncogene family, which is implicated in the progression of certain cancers. Loss of function of let-7 is followed by an increase in RAS protein levels; however, studies showed that reintroduction of let-7 mimics inhibits the propagation of carcinogen cells and decreases the growth of existing lung cancers (82). Similarly, in colon and hepatic cancer cells, miR let-7 mimics were also found to elevate cell apoptosis, downregulate cell proliferation, and invasion (76).

Likewise, in order tore-establish miR-34 expression in the cancer cells, miR-34 mimic was delivered systemically to mice lung tumor, leading to cell death by acting on different types of molecules like MAPK, Bcl-2, and p53 (76). Other miRNA mimics, found to restore the purpose of tumor suppressions, are miR-16, miR-21, and miR-26a (83). miRNA mimics are particularly useful for figuring out how tumor suppressor miRNAs contribute to cancer and are also essential for evaluating miRNA function. By restoring these miRNAs’ activity, scientists can discover their therapeutic potential and investigate cutting-edge approaches to treating cancer with targeted miRNA replacement therapy (84).

Conversely, miRNA mimics face challenges related to delivery mechanisms, constancy or stability in the blood and off target effects. miRNA structures can activate innate immune responses through pattern recognition receptors like Toll-like receptors, Melanoma Differentiation-Associated protein 5, leading to release of cytokines causing inflammation. Therefore, chemical modifications like 2’-OMe or 2’-MOE and appropriate delivery systems such as liposomes and nanoparticles are getting developed to deliver efficiently miRNA mimics to the tumor site minimizing systemic toxicity and overcoming the barriers of immunostimulation (7, 21).

## 7 Challenges in the miRNA therapeutics

A wide range of clinical studies suggests the extensive potential of miRNA therapeutics, on the other way miRNA based drugs have encountered crucial obstacles in clinical translation (Tables 3, 4). Only few miRNA based therapeutic drugs have fulfilled the clinical trials and most of the drugs have been discontinued due to safety concerns (85). The first miRNA-based drug to enter clinical testing was Miravirsen, an antagomiR targeting miR-122 for hepatitis C virus infection. Miravirsen demonstrated strong antiviral efficacy and advanced to multiple Phase II clinical trials (86). In oncology, MRX34, a liposomal miR-34 mimic, was the first miRNA-based cancer therapeutic to achieve clinical evaluation. Although early signs of antitumor activity were observed in melanoma, hepatocellular carcinoma, non-small cell lung cancer, and renal carcinoma, the Phase I trial was terminated because of severe immune-related adverse events (85). These discontinuities emphasizes that the miRNA based therapies have not yet gained a critical clinical breakthrough.

**Table 3:** Clinical validation and translational status of selected miRNAs.

miRNA	Function as	Cancer type	Level of clinical validation	Clinical trials	References
miR-21	OncomiR	Breast, lung, colorectal	Strong diagnostic and prognostic biomarker	miR-21 has not completed advanced oncology trials	(7, 111)
mR-155	OncomiR	CLL, lymphoma, breast	Strong validation in hematologic malignancies; correlated with disease progression and survival	Cobomarsen (MRG-106) in Cutaneous T-Cell Lymphoma and related hematological malignancies	(111)
miR-17–92 cluster	OncomiR cluster	Lung, lymphoma, colorectal	Strong mechanistic and prognostic validation	Cluster has not completed trials yet	(112)
let-7 family	Tumor suppressor	Lung, breast, melanoma	Strong biological and prognostic validation	No clinical therapeutic achieved yet due to challenges in delivery, stability and off target affects	(113)
miR-34	Tumor suppressor	Colorectal, ovarian, melanoma	Strong preclinical validation; p53-regulated tumor suppressor	MRX34 mimic, discontinued later on	(114)
miR-122	Tumor suppressor	Hepatocellular carcinoma	Strong validation in liver biology; prognostic in HCC	Miravirsen, RG-101 (HCV trials)	(115)

**Table 4:** Clinical evaluation of miRNA-based therapeutics across multiple diseases.

miRNA Drug	Targeted miRNA	Mode of Action	Disease	Mode of delivery	Phase	References
miR-10b	miR-10b	miR-10b in Diagnosis and *in vitro* Investigation of Anti-miR-10b Therapeutics	Glioblastoma, Brain tumors, Oligodendroglioma	Nano-particle assisted delivery	Phase I recruiting	(85, 116)
MesomiR	miR-16	miRNA mimic	Non-small lung cancer	Bacterial minicell nanoparticles	Phase I completed	(7, 85, 116)
MRX34	miR-34a	miRNA mimic	Hepatocellular carcinoma, melanoma, Lymphoma, multiple myeloma	Liposomal nanoparticle delivery system	Phase I	(85, 117)
MRG-106	miR-155	Anti-miR	Cutaneous T-cell Lymphoma, Chronic Lymphocytic Leukemia	Chemical modification (LNA)	Phase I completed	(116)
Serum microRNA-25	miR-25	miR-25 as diagnostic	Pancreatic cancer	Small extracellular vesicles	Under trial	(7, 116)
miR-10	miR-10	Anti-miR-10	Glioma	Nanoparticles	Phase I recruiting	(85, 116)

Secondly, here efficient and safe delivery of the therapeutics is crucial for the successful miRNA therapy. Studies show that miRNA mimics and inhibitors are highly susceptible to nuclease degradation and reveal short half-lives in circulation, restricting the administration primarily to intravenous or subcutaneous routes (86). However, nanoparticles based delivery of miRNA targets to amplify the healing effectiveness but most of the systems have so far to attain their testing in humans. Therefore the challenge is to create efficient and compatible carrier systems for miRNA therapy.

Regulatory and economic barriers also contribute to the slow progress of miRNA-based therapeutics. A regulatory gap persists for nanomedicines, with limited standardized guidelines for quality control, manufacturing processes, and safety evaluation (85, 87). The structural and physicochemical complexity of nanoformulations complicates large-scale production and compliance with good manufacturing practices. Furthermore, the high cost of miRNA-based drugs and advanced delivery systems raises concerns regarding cost–benefit balance compared with existing therapies (85).

## 8 Conclusion and future prospects

miRNAs have emerged as important biomarkers for the prognosis of diseases like cancer and diagnosis, and are also revolutionizing the understanding of post-translational gene regulation. The 1st miRNA, lin-4, was discovered in 1993, and subsequent advances in miRNA research, supported by technologies such as microarrays, NGS, and qRT-PCR, have enabled high-throughput expression profiling of miRNAs. Studies indicate that miRNAs possess a critical role in regulating numerous physiological processes, such as differentiation, apoptosis, and the development of organisms. miRNAs are known as mutated genes (oncogenes) or tumor suppressors based on their upregulation or downregulation in several cancers. miRNAs have also emerged as a useful tool in prognosis, therapeutic interventions, and diagnosis, and are considered among the most efficient biomarkers for the recognition of different types of cancers in the tissues as well as in body fluids.

Various miRNA-based curatives have been developed, such as inhibition and replacement therapies, which are utilized for the suppression of cancers. However, future studies are required in the field of miRNA therapies by improving the delivery systems, reducing off-target effects without activating the immune system against the mimics or inhibitors, and expanding clinical applicability. With continuing scientific and technological advancements, miRNA therapeutics may become an essential component of next-generation oncology protocols, providing novel solutions for the improvement of outcomes in advanced-stage cancers. Therefore, continued research is a key to conquering the hurdles and efficiently making use of miRNA in early disease detection, prognosis, and treatment.
